# Efficacy and safety of non‐cross‐linked hyaluronic acid compound in the treatment of keratosis pilaris: A split‐body randomized clinical trial

**DOI:** 10.1111/jocd.16532

**Published:** 2024-08-23

**Authors:** Yao Li, Shi‐Wei Wang, Yan‐Hua Liu, Mu‐Yan Zou, Jia‐Xu Wu, Sheng‐Kang Luo, Wei‐Jin Hong

**Affiliations:** ^1^ Department of Plastic and Reconstructive Surgery Guangdong Second Provincial General Hospital Guangzhou Guangdong China; ^2^ Medical Department Imeik Technology Development Co., Ltd Beijing China

**Keywords:** clinical efficacy, keratosis pilaris, non‐cross‐linked hyaluronic acid compound

## Abstract

**Background:**

Keratosis pilaris (KP) is a prevalent benign dermatological condition characterized by small bumps at the hair follicles alongside surrounding redness, significantly impacting both aesthetics and mental well‐being.

**Objective:**

This study investigated the potential benefits of a non‐cross‐linked hyaluronic acid (HA) compound for treating KP.

**Methods:**

A split‐body, investigator‐blinded, randomized, intraindividual comparative clinical trial was conducted. The non‐cross‐linked HA compound was injected into KP‐affected regions on both upper arms. The treatment was delivered across four sessions scheduled at 4‐week intervals. Blinded physicians and patients assessed differences in erythema, skin roughness, and overall scores between treated and control areas at the final follow‐up visit. At the 12th and 24th weeks post‐treatment, a four‐point scale was utilized to assess subjects' perceived treatment efficacy. Additionally, dermoscopic images, histological alterations, and adverse events were monitored.

**Results:**

Physician assessments revealed a significant reduction in roughness and overall scores for treated areas compared to controls. Patient self‐assessments also reflected improvements in roughness, redness, and overall scores for treated sides at the final visit, with 35.71% of patients demonstrating sustained improvement in redness and 71.43% reporting persistent improvements in roughness at 24th weeks post‐treatment. The dermatoscopic examinations revealed a notable enhancement in both the quantity of follicular plugs and the extent of erythema among the subjects in the treatment group. Histopathological outcomes also demonstrated improvement.

**Conclusion:**

This study suggests that the non‐cross‐linked HA compound effectively improves skin roughness and promotes hair shaft growth in KP treatment, demonstrating a favorable safety profile. These findings position it as a potentially viable alternative therapy in clinical practice.

## 1 INTRODUCTION

Keratosis pilaris (KP) is a common, harmless hyperkeratotic condition distinguished by follicular papules accompanied by varying levels of perifollicular erythema and hyperpigmentation. These skin abnormalities are predominantly observed on the extensor surfaces of the upper and lower limbs, although they can also manifest on the face, buttocks, and trunk.^1^ While traditional and alternative topical treatments, such as moisturizers, peeling agents, IPL treatments, and laser interventions, have shown effectiveness in treating KP, the exploration for innovative therapeutic approaches continues.^2^ Notably, KP lesions tend to improve during summer months and with consistent emollient use, suggesting potential benefits of improved skin hydration and increased ambient humidity.^3^

Hyaluronic acid (HA) is a widely distributed glycosaminoglycan crucial for regulating skin moisture balance. Beyond its well‐established applications in facial volumization and wrinkle reduction, recent times have seen injectable HA formulations being studied for skin augmentation to improve skin texture and quality overall.^4^ Non‐cross‐linked HA stands out for its excellent skin hydrating benefits, providing moisture without the volumizing effects associated with cross‐linked HA fillers.^5^ While the use of small‐particle HA for skin rejuvenation has not been investigated in the context of KP, existing treatment options for this condition often yield limited success. Research has revealed that in the lesions of various dermatological conditions, such as psoriasis, the typical reticular structure of hyaluronic acid (HA) is partially absent in the spinous and granular layers.^6^ Furthermore, HA exhibits potential in promoting keratinocyte migration, enhancing wound healing, and facilitating barrier repair. This study aimed to evaluate the efficacy of non‐cross‐linked HA compound dermal injections to enhance the appearance and texture of KP lesions on the upper extremities using a skin‐boosting approach, exploring its potential benefits for treating KP and offering additional treatment options.

## 2 METHODS

### 2.1 Study design

This was an investigator‐blinded, split‐body, randomized, intraindividual comparative clinical trial with study protocol approval from the Ethical Review Committee. The research proceeded with written consent obtained from all participants.

### 2.2 Patient selection

Participants in the study were enlisted through the Plastic and Reconstructive Surgery Department. Inclusion criteria comprised healthy individuals aged 18 years or older with confirmed bilateral upper extremity KP. Exclusion criteria encompassed a history of keloid or hypertrophic scarring, pregnancy, lactation, active skin diseases, malignant neoplasms, exposed sores, and infections at any cutaneous location. Additionally, Patients undergoing KP treatment within the past 6 months or using topical medications or emollients within the previous month were excluded.

### 2.3 Patient randomization

A computer‐generated random number table was utilized to assign a unique number to each patient, subsequently used for randomization into one of two groups. Patients assigned odd numbers were treated on their left upper extremity, while their right upper extremity served for comparison. In contrast, patients assigned even numbers underwent treatment on their right upper extremity, while their left upper extremity served for comparison. All treatments were administered by the same clinician in the study.

### 2.4 HA injectable formulations and treatment

Topical anesthesia (Compound Lidocaine Cream®, TongFang Pharmaceutical Group Co., Ltd.) was applied to the designated treatment area for 45 min prior to the procedure. Routine disinfection protocols were followed before treatment commencement. The non‐cross‐linked HA compound (Hearty®, Imeik Technology Development Co., Ltd.) features a blend of main ingredients including sodium hyaluronate, proline, glycine alanine, L‐carnosine, and vitamin B_2_. This product, which had been previously sanctioned by the Chinese National Medical Products Administration for rejuvenating neck wrinkles, was administered under sterile conditions, targeting the lesions on the extensor side of the upper limb with multiple microinjections into the subcutaneous layer, with injection points spaced 0.5 cm apart, administering 0.025 mL per injection. The treatment regimen consisted of four sessions spaced at 4‐week intervals.

### 2.5 Study procedures and follow‐up

Patients independently evaluated the severity of redness (erythema) and roughness on each arm using a standardized scale ranging from 0 (least severe) to 3 (most severe). Each arm could achieve a maximum score of 6. After randomization, patients underwent a series of four injections of the non‐cross‐linked HA compound in one upper extremity, which was identified as the treatment site, while the contralateral arm served as the control group, remaining untreated. Injections were administered at 4‐week intervals. Four weeks after the final injection, patients again assessed erythema and roughness using the same scale. Additionally, two blinded physicians independently evaluated these features on each arm using the same scale during the final visit. Standardized digital photographs and dermoscopic images were captured at baseline and the final visit within the same clinic room to ensure consistency. Histological examinations were performed on 1.0‐mm punch biopsy specimens obtained from three patients.

### 2.6 Outcome measures

The crux of the evaluation focused on elucidating disparities between the treatment and control sites, constituting the primary outcome measure. These disparities were assessed by evaluators in terms of erythema (redness), roughness, and overall scoring. To ensure consistency, two blinded physicians received training on the standardized assessment scale employed in the study. These physicians independently evaluated a set of archived skin images using the same qualitative subscales. Any discrepancies in scoring were resolved through discussions until consensus was reached. Secondary endpoints included patient self‐assessment scores using the same standardized scale, standardized digital clinical photographs, dermoscopic images, and histological changes. Additionally, at the 12th and 24th weeks post‐treatment, subjects' perceived efficacy was evaluated utilizing a four‐point Likert scale (0 = significant improvement, 1 = moderate improvement, 2 = mild improvement, 3 = no improvement or deterioration). The improvement rate was characterized by the proportion of respondents scoring between 0 and 2 on the questionnaire, thus facilitating the assessment of long‐term effectiveness in evaluating erythema and roughness. Throughout the study, meticulous documentation was diligently conducted on any adverse physical manifestations associated with the injections (e.g., redness, swelling, discomfort, itching, and bruising). Subsequently, the incidence rate was calculated to assess the safety profile of the non‐cross‐linked HA compound for the treatment of KP.

### 2.7 Statistical analysis

The comparison of improvement levels between the treatment and control groups for both blinded physician ratings (evaluating erythema, roughness/bumpiness, and overall score) and patient self‐ratings (evaluating erythema, roughness/bumpiness, and overall score) was conducted using the Wilcoxon signed‐rank test.

## 3 RESULTS

### 3.1 Patient baseline demographic characteristics

Fifteen individuals diagnosed with keratosis pilaris and exhibiting bilaterally symmetrical lesions qualified and were included in the study. The data analysis ultimately included 14 participants who successfully completed the study. Patient demographics are detailed in Table 1.

**TABLE 1:** Participant demographics of the study.

Characteristics	No./total no.
Completed study	14/15
Dropped out	1/15
Sex	
Male	4/14
Female	10/14
Age on set (years)	
10–19	1/14
20–29	12/14
30–49	1/14
Fitzpatrick skin type	
II	2/14
III	8/14
IV	4/14
Itch	
Yes	6/14
No	8/14
Family history	
Yes	11/14
No	3/14

### 3.2 Physician assessment scores

**FIGURE 1:**
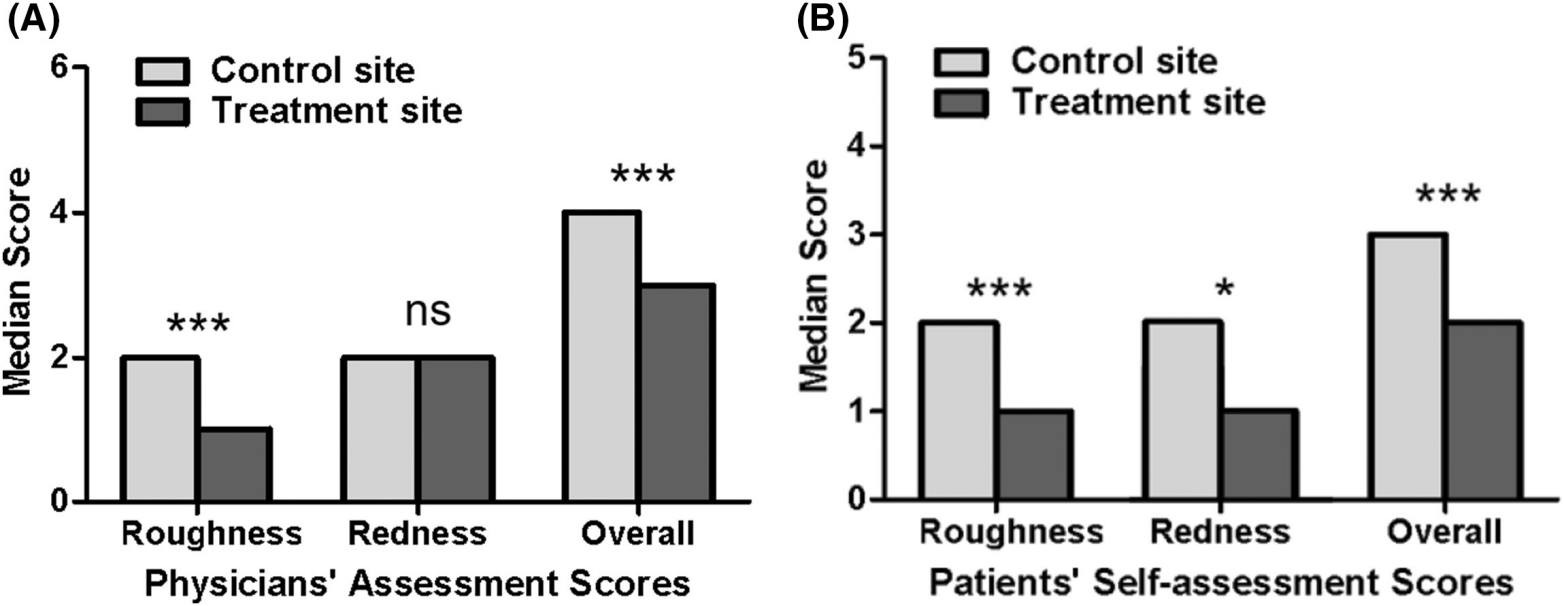
The outcome measures for both the treatment and control sites were compared at the last follow‐up visit. (A) Physicians' assessment scores. The roughness and overall scores for treatment sides demonstrated a notable reduction compared to the control sides (*p* < 0.05). (B) Patients' self‐assessment scores. The roughness, redness, and overall scores for treatment sides exhibited a decrease in comparison to the control sides (*p* < 0.05).

At the final follow‐up visit, blinded physicians assigned a median roughness/bumpiness score of 1.0 (IQR, 1–2) to the treatment side compared to 2.0 (IQR, 2–3) for the control side, demonstrating a statistically significant difference of 1 point (*p* < 0.05) (Figure 1A). The median physician‐assigned erythema score was the same (2.0; IQR, 1–2) for both treatment and control sides. However, the median total score was 3.0 (IQR, 2–3) for the treatment sides and 4.0 (IQR, 3–4) for the control sides, with a statistically significant difference of 1 point (*p* < 0.05) (Figure 1A).

### 3.3 Patient self‐assessment scores

**FIGURE 2:**
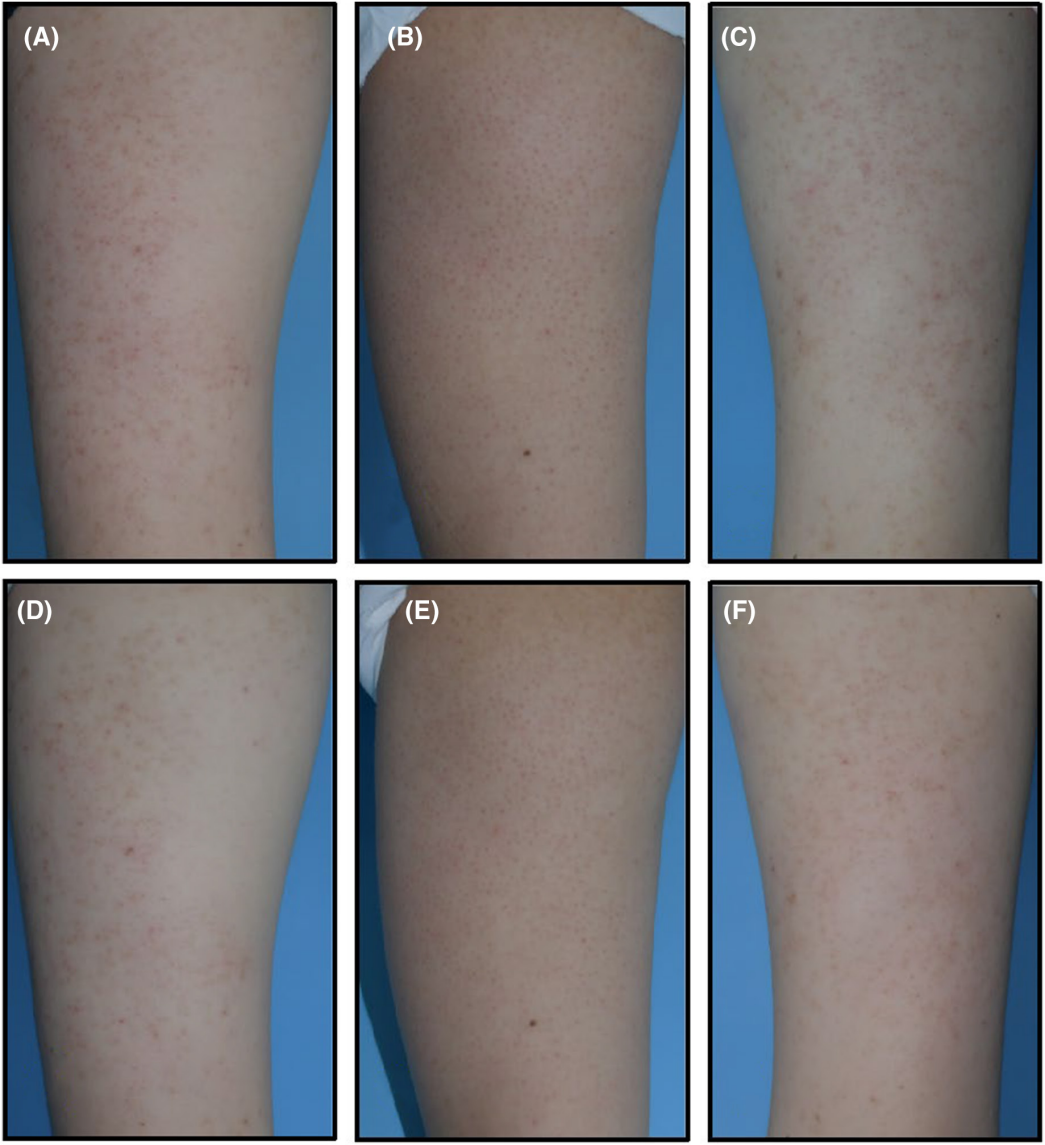
The clinical photographs from the baseline and the final visit. (A, D) A KP patient's photographs at the baseline (A) and at the final visit (D). (B, E) Another KP patient's photographs at the baseline (B) and at the final visit (E). (C, F) One more KP patient's photographs at the baseline (C) and at the final visit (F).

Before starting treatment, there were no significant differences in the median scores reported by patients for roughness/bumpiness, erythema, and total score between the treatment and control sides. At the final visit, the median patient‐reported roughness/bumpiness score was 1.0 (IQR, 1–2) for the treatment side compared to 2.0 (IQR, 2–3) for the control side (*p* < 0.05) (Figure 1B). Similarly, the median patient‐reported erythema score was 1.0 (IQR, 1–2) for the treatment side and 2.0 (IQR, 1–2) for the control side (*p* < 0.05). The median overall score on the treatment side was 2.0 (IQR, 2–4) compared to 3.0 (IQR, 3–5) on the control side, demonstrating a statistically significant difference of 1.0 point (*p* < 0.05) (Figure 1B). Representative photographic images of KP patients were shown in Figure 2 (a‐f).

### 3.4 Skin imaging

**FIGURE 3:**
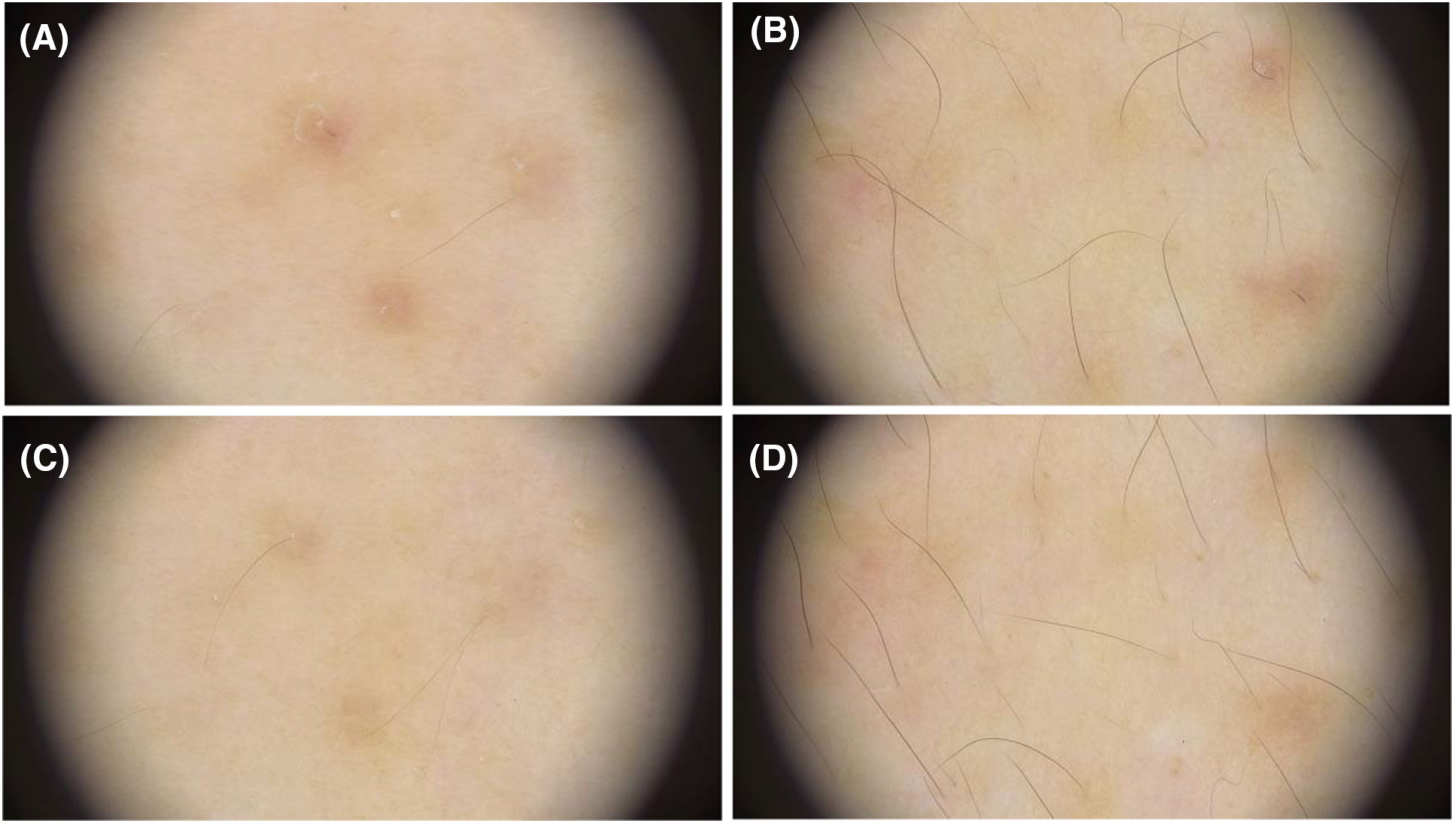
Dermoscopic image (20×) of KP at the baseline (A, B) and at the final visit (C, D). Significant improvements in hyperkeratosis, hyperpigmentation, and perifollicular erythema were noted by the final visit, with fewer coiled or looped hairs and more normal hairs observed.

Dermoscopic images revealed significant improvement. Following the final treatment session, the count of follicular plugs on the treatment sides showed a 73.3% improvement. The extent of perifollicular erythema demonstrated a 20.0% improvement on the treatment sides, while hyperpigmentation showed minimal improvement (Figure 3).

### 3.5 Histopathological examination

**FIGURE 4:**
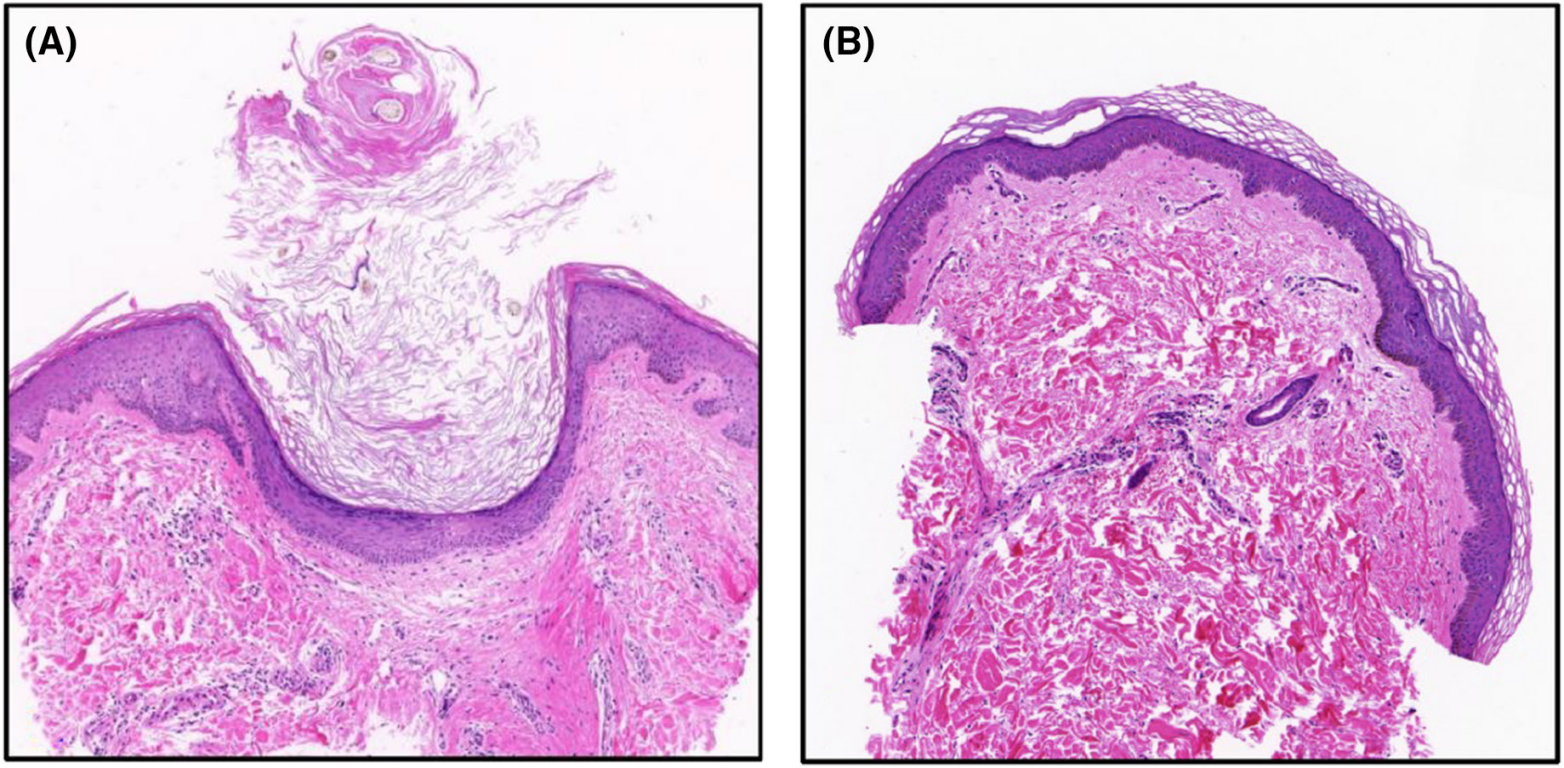
Histopathologic changes (200×) of KP at the baseline (A) and at the final visit (B). At baseline, there existed follicular infundibular dilatation, marked by the occlusion of follicular lumina with a keratinous plug accompanied by localized peri‐infundibular parakeratosis. Additionally, an intricate interplay of basket weave and lamellated orthokeratosis was evident, alongside perifollicular lymphocytic infiltrate. All aforementioned pathological manifestations showed improvement by the final visit.

Histopathological examination at baseline revealed hyperkeratosis, prominent follicular plugging, and lymphocytic infiltration, consistent with the clinical diagnosis of KP. All pathological features demonstrated improvement at the final visit (Figure 4).

### 3.6 Subject questionnaire evaluation

We evaluated the enhancement in erythema and roughness at 12 and 24 weeks post‐treatment by the subjects' perceived treatment efficacy questionnaire. This assessment was based on the subjects' subjective experiences. At 24 weeks post‐treatment, the improvement rate for erythema on the treated side had attained 35.71%, and for roughness, it had attained 71.43%. These results indicated that subjects perceived a continued improvement in skin roughness, however, the improvement in erythema was limited (Table 2).

**TABLE 2:** Patient questionnaire evaluation *N* (%).

Follow‐up time		*N*	Significant improvement	Moderate improvement	Mild improvement	No improvement or deterioration	Improvement rate
12 week post‐treatment	Redness	14	0 (0.00%)	3 (21.43%)	3 (21.43%)	8 (57.14%)	42.86%
	Roughness	14	0 (0.00%)	5 (35.71%)	6 (42.86%)	3 (21.43%)	78.57%
24 week post‐treatment	Redness	14	0 (0.00%)	3 (21.43%)	2 (14.29%)	9 (64.29%)	35.71%
	Roughness	14	0 (0.00%)	3 (21.43%)	7 (50.00%)	4 (38.57%)	71.43%

### 3.7 Adverse events

No unexpected adverse events were observed during the research period. Two (14.3%) patients experienced transient post‐inflammatory hyperpigmentation (PIH). Moreover, no adverse symptoms were recorded in the subjective questionnaires of the participants at 12 and 24 weeks post‐treatment.

## 4 DISCUSSION

Keratosis Pilaris (KP) is a widely inherited dermatological condition typically presenting in childhood.^7^ Many patients in our study reported feeling self‐conscious about their upper arms, especially during warm weather when wearing short sleeves. This study explored the efficacy of injectable hyaluronic acid (HA) formulations in the management of KP. Our findings demonstrated significant improvements in skin roughness after a course of three treatments spaced 4 weeks apart, as assessed by both blinded physicians and patients themselves, which were maintained for up to 24 weeks. Notably, erythema (redness) showed minimal improvement.

To address various aspects of keratosis pilaris lesions, therapeutic approaches such as moisturizers, exfoliants, and laser therapies are commonly utilized. However, these interventions often exhibit limited and temporary effectiveness. In numerous instances, general skincare routines focused on preventing dryness are essential. The development of a protective film on the skin through prolonged emollient application minimizes water loss and boosts hydration, thereby improving lesions. These agents also fill gaps between skin cells, leading to a softer texture.^8^ However, the tedious and time‐consuming nature of daily emollient application can deter patients from adhering to consistent treatment schedules.

HA is an endogenous glycosaminoglycan inherently embedded within the intricate network of the extracellular matrix, notably prevalent in connective, epithelial, and neural tissues microenvironments. It assumes a pivotal role across multifarious biological processes, encompassing wound healing, inflammatory responses, angiogenesis, and the orchestration of embryonic development.^9^ HA is essential for maintaining skin hydration, elasticity, structure, and firmness, leading to a softer, smoother appearance. Due to its non‐toxic and non‐sensitizing properties, HA is a popular ingredient in various skincare products designed to enhance overall skin quality.

In recent years, injectable HA formulations have been used to improve skin quality. Unlike emollients which primarily target the stratum corneum (outermost skin layer), HA injections reach both the stratum corneum and the dermis (deeper layer). By providing hydration and creating a stable extracellular matrix essential for fibroblast function, HA injections offer a promising approach.^10^ The simplicity, quickness, and non‐invasive nature of the injection process, coupled with HA's exceptional water‐binding capacity, are driving the increasing popularity of injectable HA formulations for skin rejuvenation.^11^

Our study augments the extant corpus of research concerning HA within dermatological contexts, which had traditionally centered on evaluating global skin attributes, particularly pertaining to aging phenomena. To the best of our knowledge, this investigation represents the inaugural endeavor in investigating the efficacy of non‐cross‐linked HA compounds for the management of KP. Furthermore, our findings provide the first evidence for the effectiveness of injectable non‐cross‐linked HA compound in addressing the roughness and textural abnormalities associated with KP. These results suggest that non‐cross‐linked HA offers a promising and effective treatment option for non‐erythematous (non‐reddened) variants of KP.

The selection of the non‐cross‐linked HA formulation containing L‐carnosine, vitamins, and trace amino acids was grounded in specific theoretical considerations. When the hydration levels of the stratum corneum (SC), the outermost layer of the skin, falls below normal levels, the function of enzymes crucial for normal skin desquamation (flaking) becomes impaired. This impairment leads to corneocyte (skin cell) binding and buildup on the outer layer of the skin, resulting in a dry and rough appearance.

HA is renowned for its capacity to furnish hydration and structural reinforcement to the dermis, the deeper layer of the skin. Nonetheless, research posits that HA also resides within the epidermis, the outermost layer, where it potentially contributes to moisturizing the SC and modulating its barrier function.^12^ Additionally, HA has the ability to engage with CD44, a crucial receptor located on the surface of epidermal keratinocytes, thereby orchestrating epidermal differentiation and modulating lipid synthesis/secretion.^13^

The utilization of non‐cross‐linked HA compound as a supplementary therapy fosters comprehensive enhancement of skin quality and heightened hydration levels. L‐carnosine, a dipeptide, provides supplementary advantages by mitigating the generation of free radicals and advanced glycation end products (AGEs).^14^ It also protects HA from degradation caused by free radical processes.^15^ Glycine, alanine, and proline were included as natural moisturizing factors and pH regulators. This combination of ingredients makes non‐cross‐linked HA compound an excellent choice for treating KP by simultaneously moisturizing the skin, softening the outer layer (cutin), and providing essential nutrients.

The prevailing theory posits that KP originates from aberrant keratinization within the follicular epithelium, culminating in the development of a keratinous plug within the follicular infundibulum.^16^ However, dermoscopic examination has led to the alternative hypothesis that KP may be primarily a hair shaft disorder rather than a keratinocyte dysfunction. This viewpoint posits that irregular or curved hair shafts breach the follicular epithelium, instigating an inflammatory response and subsequent anomalous keratinization associated with KP.^17^ The histological staining results revealed a diminution in follicular occlusion following treatment. Additionally, there was a certain degree of improvement in hyperkeratosis and inflammatory responses relative to pre‐treatment conditions.

Our dermoscopic findings support this notion. After four treatment sessions spaced 3 weeks apart, we observed the development of vellus hair (fine, soft hairs) in the treated areas under dermoscopy, which was not as evident in photographs. These findings align with the observed histological changes. This suggests that the non‐cross‐linked hyaluronic acid compound may improve KP by nourishing the hair shaft and promoting normalized hair growth. The administration of the non‐cross‐linked HA compound was well‐tolerated, with no instances of serious or unforeseen adverse events reported. Transient post‐inflammatory hyperpigmentation was the most commonly observed side effect, but it completely resolved over time. Individuals with keratosis pilaris exhibit varying degrees of anomalies in keratinocyte metabolism or compromised barrier function, rendering their skin more susceptible to external stimuli.^7^ The mechanical trauma induced by therapeutic microneedling activates the cutaneous defense mechanisms, precipitating the release of copious inflammatory mediators to attenuate tissue damage. During the inflammatory cascade, the skin discharges a plethora of inflammatory mediators and cytokines, such as interleukins and tumor necrosis factor, which augment melanocyte activity, thereby potentiating melanin synthesis in the damaged locale.^18^ This phenomenon represents a protective response by the skin to external insults. However, this response is transiently observed post‐treatment, with subsequent inflammatory effects being mitigated through the synergistic action of constituents like hyaluronic acid and L‐carnosine. Additionally, sun protection during the treatment period may further minimize the risk of post‐inflammatory hyperpigmentation.

This study highlights the potential of non‐cross‐linked HA compound injections as an alternative treatment for KP. Nevertheless, constraints are evident, encompassing the abbreviated duration of follow‐up, the diminutive size of the patient sample, and the absence of data for objective efficacy assessment. Further investigations involving larger cohorts of patients and protracted follow‐up intervals are imperative to comprehensively elucidate the enduring efficacy of injections containing non‐cross‐linked HA for managing KP. Additionally, advanced non‐invasive skin evaluation techniques are utilized to quantitatively measure improvements in skin texture, roughness, and elasticity changes, further validating the subjective evaluation findings.

In our investigation, the administration of non‐crosslinked sodium hyaluronate complex injections for the treatment of keratosis pilaris demonstrated substantial efficacy and safety, with significant improvements observed in skin texture, roughness, and overall dermatological appearance in the treated areas, as well as in the promotion of hair shaft growth. Although further research is necessary to validate its long‐term efficacy, this study is the first to propose and investigate the potential therapeutic benefits of non‐crosslinked sodium hyaluronate complex for keratosis pilaris. These findings have significant clinical implications and may establish a novel therapeutic paradigm. Additionally, this approach may offer innovative insights and strategies for utilizing hyaluronic acid in the treatment of other dermatological conditions characterized by rough skin and hair growth abnormalities.

## AUTHOR CONTRIBUTIONS

Yao Li and Yan‐Hua Liu performed the research., Wei‐Jin Hong, Mu‐Yan Zou, and Jia‐Xu Wu designed the research study. Sheng‐Kang Luo and Shi‐Wei Wang contributed essential reagents or tools. Yao Li, Yan‐Hua Liu and Jia‐Xu Wu analyzed the data. Yao Li and Wei‐Jin Hong wrote the paper. Each author has participated sufficiently in the work to take public responsibility for appropriate portions of the content; and agreed to be accountable for all aspects of the work. All authors have read and approved the final manuscript.

## 5 FUNDING INFORMATION

This work was supported by the Science Foundation of Guangdong Second Provincial General Hospital (TJGC‐2021016).

## CONFLICT OF INTEREST STATEMENT

The authors declare no conflicts of interest.

## ETHICS STATEMENT

The study was approved by the Guangdong Second Provincial General Hospital Medicine Ethics Committee and complied with the ethical requirements of the Declaration of Helsinki.

## Data Availability

The data that support the findings of this study are available from the corresponding author upon reasonable request.
